# Meta-analysis of the prevalence of *lymphatic filariasis* infection in mosquito vectors

**DOI:** 10.3389/fcimb.2025.1708143

**Published:** 2025-12-15

**Authors:** Tong Ye, Guang-Rong Bao, Ya Qin, Quan Zhao, Bei-Ni Chen, He Ma

**Affiliations:** 1 College of Life Science, Changchun Sci-Tech University, Shuangyang, Jilin, China; 2 College of Veterinary Medicine, Jilin Agricultural University, Changchun, Jilin, China; 3 College of Veterinary Medicine, Qingdao Agricultural University, Qingdao, Shandong, China

**Keywords:** global prevalence, lymphatic filariasis, meta-analysis, molecular diagnostics, mosquito vectors, systematic review, vector-borne diseases

## Abstract

**Background:**

*Lymphatic filariasis*, caused by *Wuchereria bancrofti*, *Brugia malayi*, and *Brugia timori*, is transmitted by mosquitoes and persists as a major neglected tropical disease. Despite extensive elimination campaigns, information on vector infection prevalence remains fragmented, hindering evidence-based vector control strategies under the World Health Organization’s Global Programme to Eliminate *lymphatic filariasis*.

**Methods:**

A systematic review and meta-analysis was performed in accordance with PRISMA guidelines. Six databases (PubMed, Web of Science, ScienceDirect, CNKI, VIP, Wanfang) were searched up to April 2025. Eligible studies reported mosquito infection rates with defined sample size, location, and diagnostic method. Study quality was appraised using the Joanna Briggs Institute checklist for prevalence studies. Statistical synthesis was conducted in R (v4.1.2) using random-effects models. Subgroup analyses and meta-regression explored heterogeneity by region, mosquito genus, and detection method.

**Results:**

Eighteen studies covering 160,423 mosquitoes from 10 countries were included. The pooled infection prevalence was 0.7% (95% CI [0.3–1.7]). Rates were highest in Asia (3.0%, 95% CI [0.0–10.7]), in *Mansonia* spp. (2.5%, 95% CI [0.8–4.9]), and when RT-PCR was applied (2.5%, 95% CI [0.0–11.0]). Higher prevalence was associated with post-2016 studies (1.9%), areas without mass drug administration programmes (1.7%), and regions with annual mean temperatures of 23–27 °C (5.2%). Considerable heterogeneity (*I*² = 100%) and publication bias (Egger’s test, *p* = 0.006) were evident.

**Conclusions:**

Filarial infections in mosquitoes remain widespread, with clear regional and methodological variability. Strengthened mosquito-based xenomonitoring, particularly using molecular diagnostic approaches, will be essential to accelerate progress toward global lymphatic filariasis elimination by 2030.

## 1 Introduction

*Lymphatic filariasis* (LF) is considered one of the significant neglected tropical diseases by the World Health Organization and is caused by parasites transmitted through mosquito bites. It is primarily attributable to three filarial species—*Wuchereria bancrofti*, *Brugia malayi*, and *Brugia timori*—and is associated with significant morbidity in endemic regions (Ichimori et al., 2014; Taylor et al., 2010; WHO, 2023). Transmission occurs through bites from infected mosquitoes, including genera *Anopheles*, *Culex*, *Aedes*, and *Mansonia*, making LF a persistent public health concern worldwide (Lourens and Ferrell, 2019; Reimer and Pryce, 2024). The prevalence and distribution of LF within mosquito populations are shaped by diverse factors, including climatic conditions, patterns of urban development, and ongoing vector management strategies, including entomological surveillance activities linked to mass drug administration (MDA) programs (Ramesh et al., 2015).

Susceptibility of different mosquito taxa to filarial infection varies across ecological contexts and geographic regions, complicating accurate modeling of disease transmission (Bhuvaneswari et al., 2023). For instance, *Culex quinquefasciatus* is frequently associated with urban settings in Africa and Asia, while *Anopheles* species are typically predominant in rural environments (Cano et al., 2014; de Souza et al., 2012). Despite numerous localized studies, comprehensive data on mosquito infection rates remain fragmented, limiting efforts to refine vector control approaches and evaluate the effectiveness of mass drug administration (MDA) programs.

According to the World Health Organization (WHO, 2023), more than 657 million individuals living in 49 countries remain vulnerable to *lymphatic filariasis*—mainly within Africa, Asia, Latin America, and the Western Pacific—remain vulnerable to *lymphatic filariasis*. Among them, around 51 million people are affected by chronic clinical outcomes such as lymphedema, elephantiasis, and hydrocele (Melrose, 2002; Rajamanickam and Babu, 2025). These debilitating manifestations not only cause severe physical impairment but also lead to social exclusion and financial hardship, reinforcing LF as a significant public health burden in endemic areas.

In response to this public health issue, the World Health Organization initiated the Global Programme for the Elimination of *lymphatic filariasis* (GPELF). The original goal aimed for elimination by 2020; nonetheless, the timeline was later extended to 2030 due to ongoing operational challenges and enduring epidemiological obstacles (Group., 2019). The program integrates mass drug administration (MDA) to disrupt transmission with morbidity management to improve the quality of life of affected individuals (Boateng et al., 2025). Since its launch, GPELF has contributed to a 74% reduction in the global LF burden, underscoring its effectiveness (Ramalingam et al., 2024). Nevertheless, obstacles remain, including the potential reappearance of LF in communities once MDA campaigns cease (Meetham et al., 2023; Santoso et al., 2020). Such instances of resurgence underline the necessity of continuous monitoring and adaptive interventions to ensure sustainable elimination.

Most previous reviews have primarily focused on LF prevalence in human hosts and associated clinical outcomes, while the entomological dimension of transmission has received limited attention (Medeiros et al., 2022). Studies investigating infection rates in mosquito vectors often show inconsistent results, largely because of differences in sampling approaches, diagnostic tools, and reporting practices (Boakye et al., 2007; Cano et al., 2014; Reimer and Pryce, 2024). Moreover, only a handful of meta-analyses have attempted to aggregate global data on LF prevalence within mosquitoes, leaving important gaps in knowledge regarding vector-specific dynamics.

The present systematic review and meta-analysis was designed to address these deficiencies by estimating worldwide infection levels of filarial nematodes in mosquito populations. We assessed published studies to quantify vector infection rates and to explore the factors influencing these variations. The results of this research enhance the clarity regarding how *lymphatic filariasis* spreads, offer evidence that can inform the creation of more focused vector control strategies, and bolster the goals of the GPELF aimed at eliminating it as a public health issue by 2030. Such improvements are anticipated to elevate health outcomes for communities residing in regions where the disease is endemic.

## 2 Method

This review and meta-analysis adhered to the 2020 recommendations set forth by the Preferred Reporting Items for Systematic Reviews and Meta-Analyses Protocols (PRISMA-P) to maintain methodological integrity and clarity (Page et al., 2021), thereby ensuring methodological transparency and reproducibility (**Supplementary Table S1**).

### 2.1 Search strategy

Databases were thoroughly searched for studies in both English and Chinese that have been published from the beginning until April 2025. A total of six databases were used: PubMed, ScienceDirect, and Web of Science (WoS) for English-language publications, while CNKI, VIP, and Wanfang were utilized for Chinese-language articles. In PubMed, the literature search employed the following strategy: (((*lymphatic filariasis*[Title/Abstract])) OR (microfilaria[Title/Abstract]))) AND (mosquito[Title/Abstract]). For ScienceDirect, the search focused on Title, Abstract, and Keywords using: (TITLE-ABSTR-KEY(*lymphatic filariasis* OR microfilaria) AND TITLE-ABSTR-KEY(mosquito) AND TITLE-ABSTR-KEY(positive rate)). Search in Web of Science using TI=(*lymphatic filariasis*) AND AB=(*lymphatic filariasis*) AND TI=(mosquitoes) AND AB=(mosquitoes). For the Chinese databases (CNKI, VIP, and Wanfang), advanced search options were employed, including synonym expansion and fuzzy matching. The primary search terms included “淋巴丝虫病” (*lymphatic filariasis*), “蚊” (mosquito), and “微丝蚴” (microfilaria).

The use of both English and Chinese databases was justified by the high disease burden of *lymphatic filariasis* in Asia, ensuring that regional epidemiological reports not indexed internationally were also captured.

### 2.2 Research selection and quality assessment

Studies were eligible if they:

1. Examined mosquito populations as the primary subject.
2. Reported positive detection of filarial nematodes.
3. Clearly documented sample size, precise sampling location, and diagnostic methods.
4. Presented data derived from a single diagnostic approach to ensure comparability.

The quality of the study was evaluated independently by two reviewers using the Joanna Briggs Institute (JBI) Critical Appraisal Checklist (specifically designed for assessing the quality of epidemiological research) (Munn et al., 2015; Porritt et al., 2014). Each item meeting the predefined criteria was assigned one point across the following indicators:

1. Sampling time explicitly reported.
2. Sampling location clearly described.
3. Sample size exceeding 1,000 mosquitoes.
4. Infection rate provided for each mosquito species. (https://gis.ncdc.noaa.gov/maps/ncei/cdo/monthly).

Publications were scored on four criteria, with one point assigned per criterion. In the evaluation of the studies, a scoring system was implemented to classify their quality. Specifically, studies that received scores ranging from 3 to 4 points were deemed to be of high quality, indicating strong evidence and reliability in their findings. On the other hand, studies that earned a score of 2 points were categorized as moderate quality, suggesting that while they held some valuable insights, they also presented certain limitations that could affect their overall conclusions. Finally, those studies that scored only 1 point were classified as low quality, raising concerns about the validity and robustness of their results. In instances where the two primary reviewers encountered differing opinions regarding study quality, a third investigator was brought into the discussion to assist in reaching a consensus and ensuring that the evaluation process remained fair and thorough. The primary reviewer (YT) supervised the assessment process to ensure methodological consistency.

Following screening and quality assessment, all relevant variables were recorded in a standardized data collection template.

### 2.3 Data extraction and analysis

Data were systematically extracted into a standardized form, including: first author, sampling year, study region, mosquito genus, sample size, number of positive samples, detection method, sampling season, and whether mass drug administration (MDA) was implemented. Variables related to the environment and geography—including latitude, longitude, altitude, average yearly temperature, and yearly precipitation—were obtained from the database of the National Climatic Data Center (NCDC) of the National Oceanic and Atmospheric Administration (NOAA).

Statistical analyses were conducted using RStudio (version 4.1.2) with the meta package (Balduzzi et al., 2019; Viechtbauer, 2010). Prior to performing the meta-analysis, four different variance-stabilizing transformations were evaluated: the natural logarithm (PLN), logit (PLOGIT), arcsine (PAS), and the double-arcsine (PFT) methods. The Shapiro-Wilk normality test was applied to guide transformation selection, with W values close to 1 and *p* > 0.05 considered indicative of normality (Balduzzi et al., 2019).

Heterogeneity among studies was assessed using Cochran’s Q, the chi-squared (*χ*²) test, and the *I*² statistic, with *p* < 0.05 and *I*² > 50% indicating significant heterogeneity (Higgins et al., 2003). Due to the considerable heterogeneity detected, pooled estimates were derived employing a random-effects model. Forest plots were generated to illustrate combined prevalence estimates and variation across studies, while publication bias was assessed through funnel plot inspection and Egger’s regression test (Egger et al., 1997).

To further investigate heterogeneity, subgroup analyses and stratified meta-regression were applied, considering the following covariates:

1. Sampling year (≤2016 vs. >2016)
2. Geographic region (Asia vs. non-Asia)
3. Season (summer vs. winter)
4. Study quality (low vs. moderate/high)
5. Mosquito genus (Culex vs. others)
6. Detection method (RT-PCR vs. other methods)
7. Mosquitoes were collected using the CDC miniature light trap (Centers for Disease Control and Prevention, Atlanta, GA, USA) or other trapping methods.
8. Implementation of MDA (Yes vs. No)
9. Mean annual temperature (23-27°C vs. other ranges)
10. Mean annual precipitation (500–1000 mm vs. other ranges).

## 3 Result

### 3.1 Search results and qualified publications

**Figure 1:**
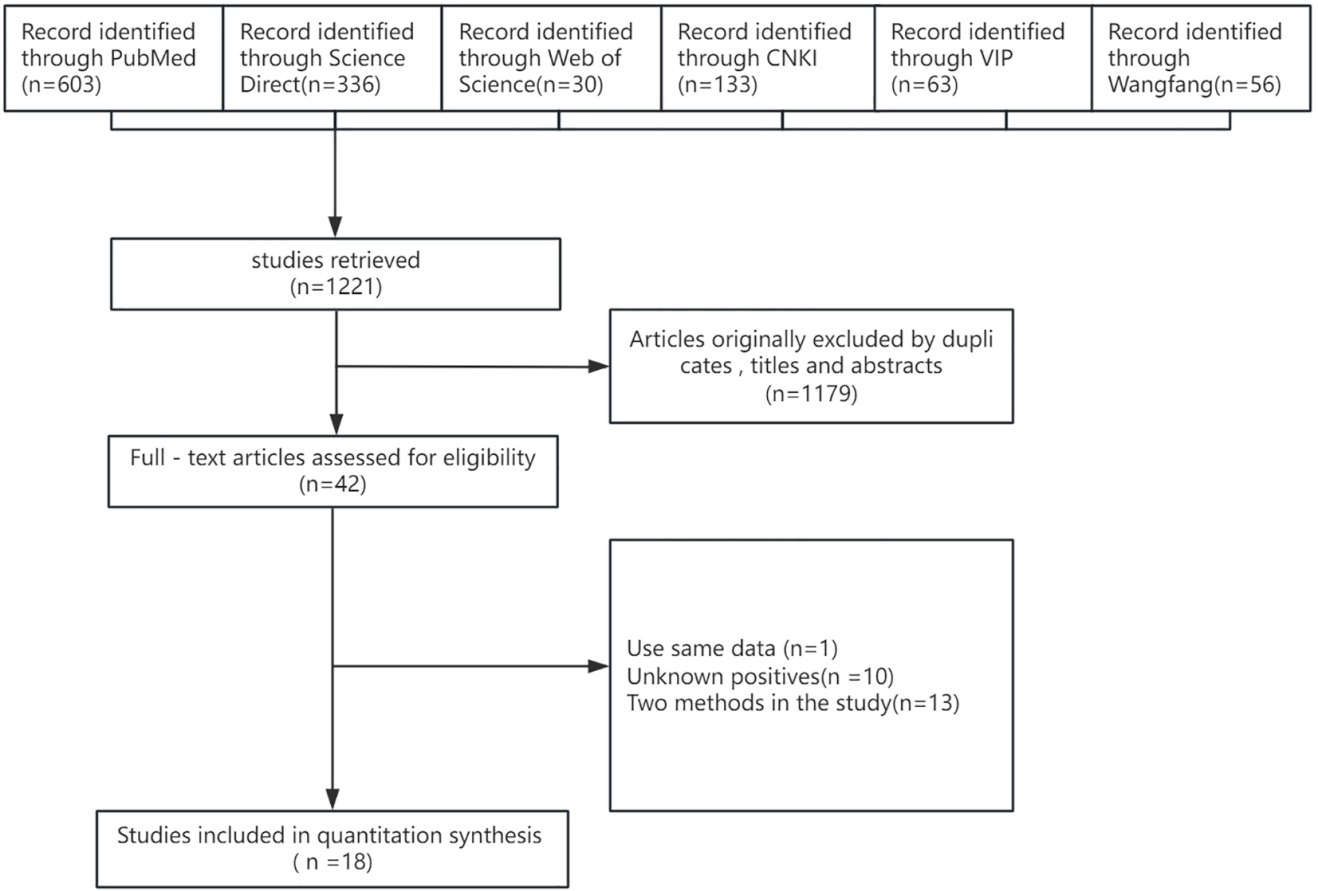
Flow diagram of literature search and selection.

The search of the database resulted in 1,221 entries. After eliminating duplicates and screening titles and abstracts, 1,179 studies were discarded. During the review of full texts, an additional 24 entries were removed: 13 employed multiple diagnostic methods with inconsistent results, 10 indicated no mosquito infections, and 1 rehashed previously published findings. Ultimately, 18 studies satisfied all eligibility requirements and were incorporated into the meta-analysis. The complete study selection process is presented in the PRISMA Flow Diagram (**Table 1**; **Figure 1**).

**Table 1:** Studies included in the analysis.

Study ID	Sampling time	County	Study quality	Detection method	No. positive/no. tested
Asian					
Vasuki et al., 2025	2017-2018	India	High	PCR	23/22,406
Abraham et al., 2024	2022-2023	India	High	RT-PCR	720/2,903
Ramalingam et al., 2024	2019	India	Middle	RT-PCR	8/680
Pilagolla and Amarasinghe 2021	2019	Sri Lanka	High	DM	5/11,321
Ridha et al., 2020	2017	Indonesia	Low	PCR	1/311
Supriyono and Tan 2020	2015	Indonesia	Middle	PCR	72/837
Oceania					
Howlett et al., 2024	2019	Samoa	High	RT-PCR	75/34,276
McPherson et al., 2022	2018-2019	Samoa	High	RT-PCR	179/42,805
Schmaedick et al., 2014	2011	American Samoa	High	PCR	54/22,014
Africa					
Bartilol et al., 2024a	2022	Kenya	High	PCR	230/1,077
Njenga et al., 2022	2012-2013	Kenya	High	RT-PCR	9/3,652
Opoku et al., 2020	2016-2017	Côte d’Ivoire	High	PCR	3/8,240
Pi-Bansa et al., 2019	2015-2016	Ghana	Middle	DM	27/1,116
Opoku et al., 2018	2017	Ghana	Middle	PCR	18/898
Dyab et al., 2016	2012-2013	Egypt	High	PCR	5/1,600
Kwansa-Bentum et al., 2014	2004	Ghana	Middle	PCR	1/564
Ughasi et al., 2012	2009	Ghana	High	PCR	8/825
Lenhart et al., 2007	1999-2001	Nigeria	High	DM	143/4,898

### 3.2 Study characteristics and quality assessment

**Tables 1**, **2** offer a summary of the methodological and epidemiological features of the studies that were included. Of the 18 eligible studies, 7 were conducted between 2004 and 2016, and 11 between 2017 and 2023. Twelve studies included sample sizes exceeding 1,000 mosquitoes. In terms of diagnostic techniques, 5 employed real-time PCR (RT-PCR), 3 used dissection microscopy (DM), and 10 used conventional PCR assays. Geographically, the studies were distributed across 10 countries, including 6 from Asia, 3 from Oceania, and 9 from Africa.

**Table 2:** Included studies and quality scores.

Reference id	No.positive/no. tested	Prevalence	Study design	Designated sampling location	Sample size exceeding 1000	Clearly defined sampling period	Reporting data integrity	Score	Study quality
Vasuki et al., 2025	23/22,406	0.10%	Cross sectional	Y	Y	N	Y	3	high
Ramalingam et al., 2024	8/680	1.18%	Cross sectional	N	N	Y	Y	2	middle
Abraham et al., 2024	720/2,903	24.80%	Cross sectional	Y	Y	Y	Y	4	high
Howlett et al., 2024	75/34,276	0.22%	Cross sectional	Y	Y	Y	Y	4	high
Bartilol et al., 2024a	230/1,077	12.07%	Cross sectional	Y	Y	N	Y	3	high
Njenga et al., 2022	9/3,652	0.25%	Cross sectional	Y	Y	Y	Y	4	high
McPherson et al., 2022	179/42,805	0.42%	Cross sectional	Y	Y	Y	N	3	high
Pilagolla and Amarasinghe 2021	5/11,321	0.04%	Cross sectional	Y	Y	Y	Y	4	high
Supriyono and Tan 2020	72/837	8.60%	Cross sectional	Y	N	Y	N	2	middle
Opoku et al., 2020	3/8,240	0.04%	Cross sectional	Y	Y	Y	Y	4	high
Ridha et al., 2020	1/311	0.32%	Cross sectional	N	N	Y	N	1	low
Pi-Bansa et al., 2019	27/1,116	2.42%	Cross sectional	N	Y	Y	N	2	middle
Opoku et al., 2018	18/898	2.00%	Cross sectional	Y	N	Y	N	2	middle
Dyab et al., 2016	5/1,600	0.31%	Cross sectional	N	Y	Y	Y	3	high
Kwansa-Bentum et al., 2014	1/564	0.18%	Cross sectional	Y	N	Y	N	2	middle
Schmaedick et al., 2014	54/22,014	0.25%	Cross sectional	N	Y	Y	Y	3	high
Ughasi et al., 2012	8/825	0.97%	Cross sectional	Y	N	Y	Y	3	high
Lenhart et al., 2007	143/4,898	2.92%	Cross sectional	Y	Y	Y	N	3	high

a Y: Yes; N: No.

c High: 4 or 3 points; Middle: 2 points; Low: 1 point.

According to the JBI quality assessment, 12 studies were rated as high quality, 5 as moderate, and 1 as low. All studies, including the low-quality report, were retained for analysis.

### 3.3 Data synthesis, heterogeneity, and robustness evaluation

**Figure 2:**
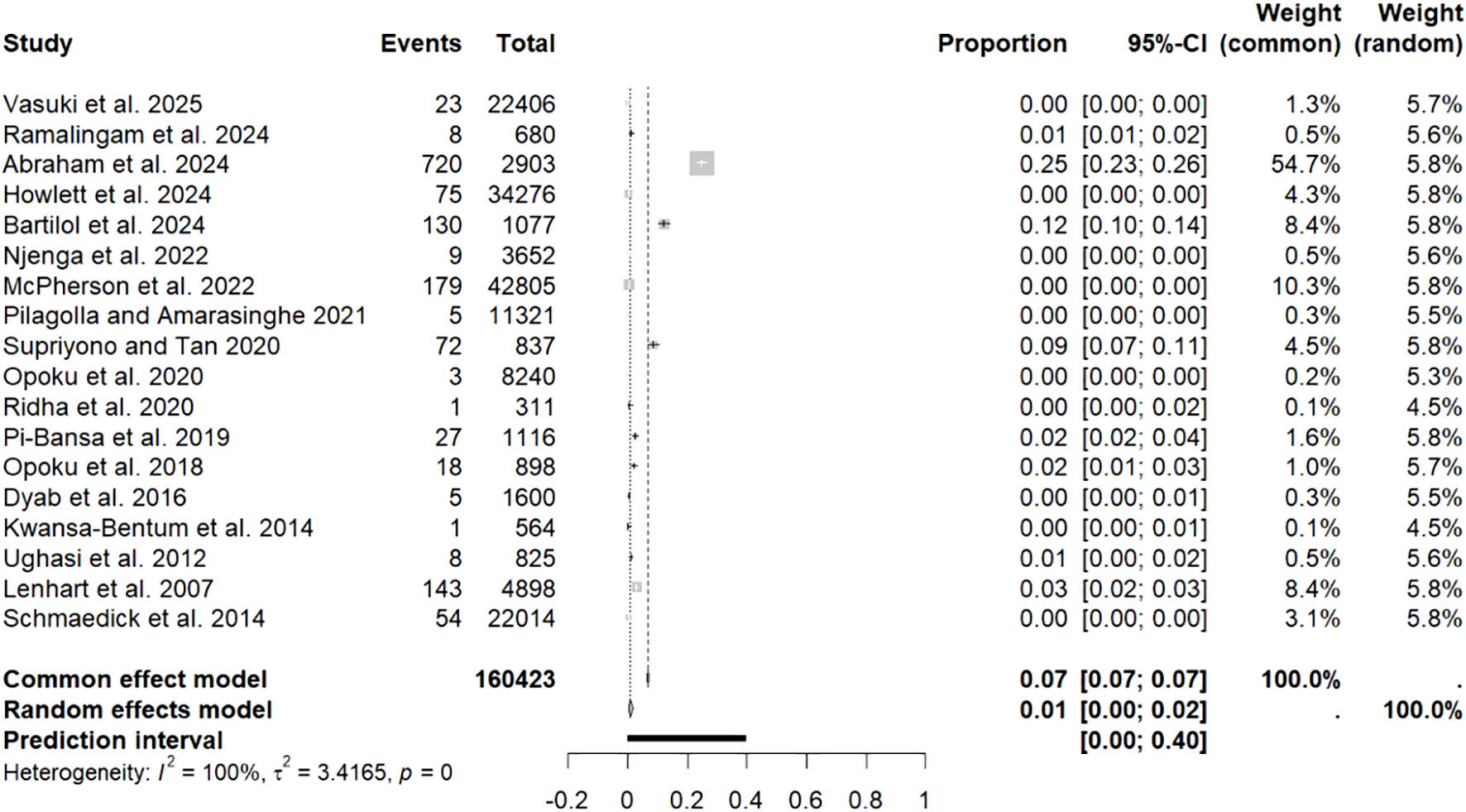
Forest plot of filarial nematodes prevalence in mosquitoes. The length of the horizontal line represents the 95% confidence interval, and the diamond represents the summarized effect.

**Figure 3:**
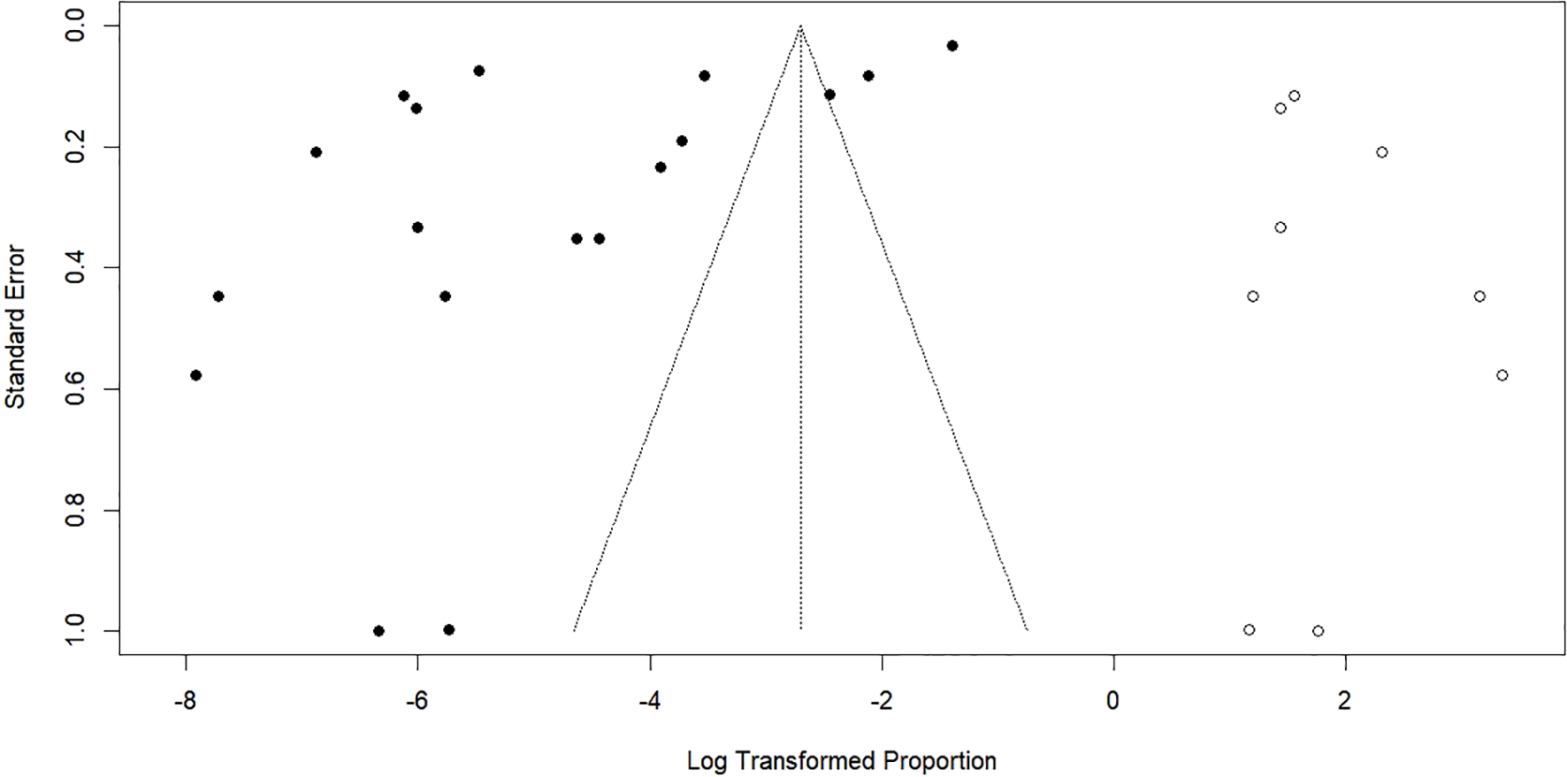
Funnel plot for the publication bias test of the included studies.

**Figure 4:**
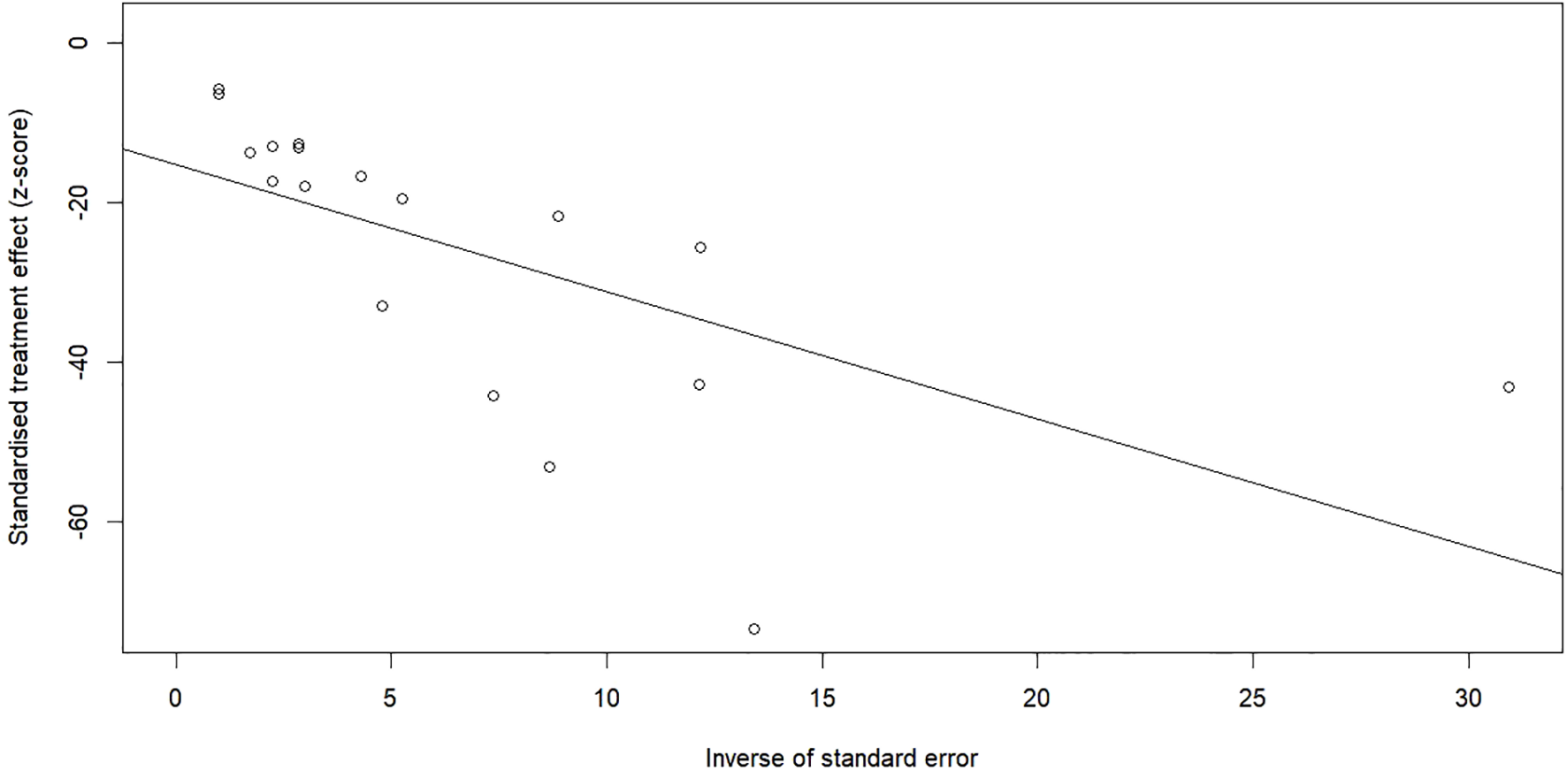
Egger’s test for publication bias.

The meta-analysis revealed significant variability (*I*² = 100%, **Figure 2**), resulting in the use of a random-effects model. Funnel plot asymmetry suggested the presence of publication bias (**Figure 3**), and this finding was statistically confirmed by Egger’s test (*p* = 0.006; **Figure 4**).

**Figure 5:**
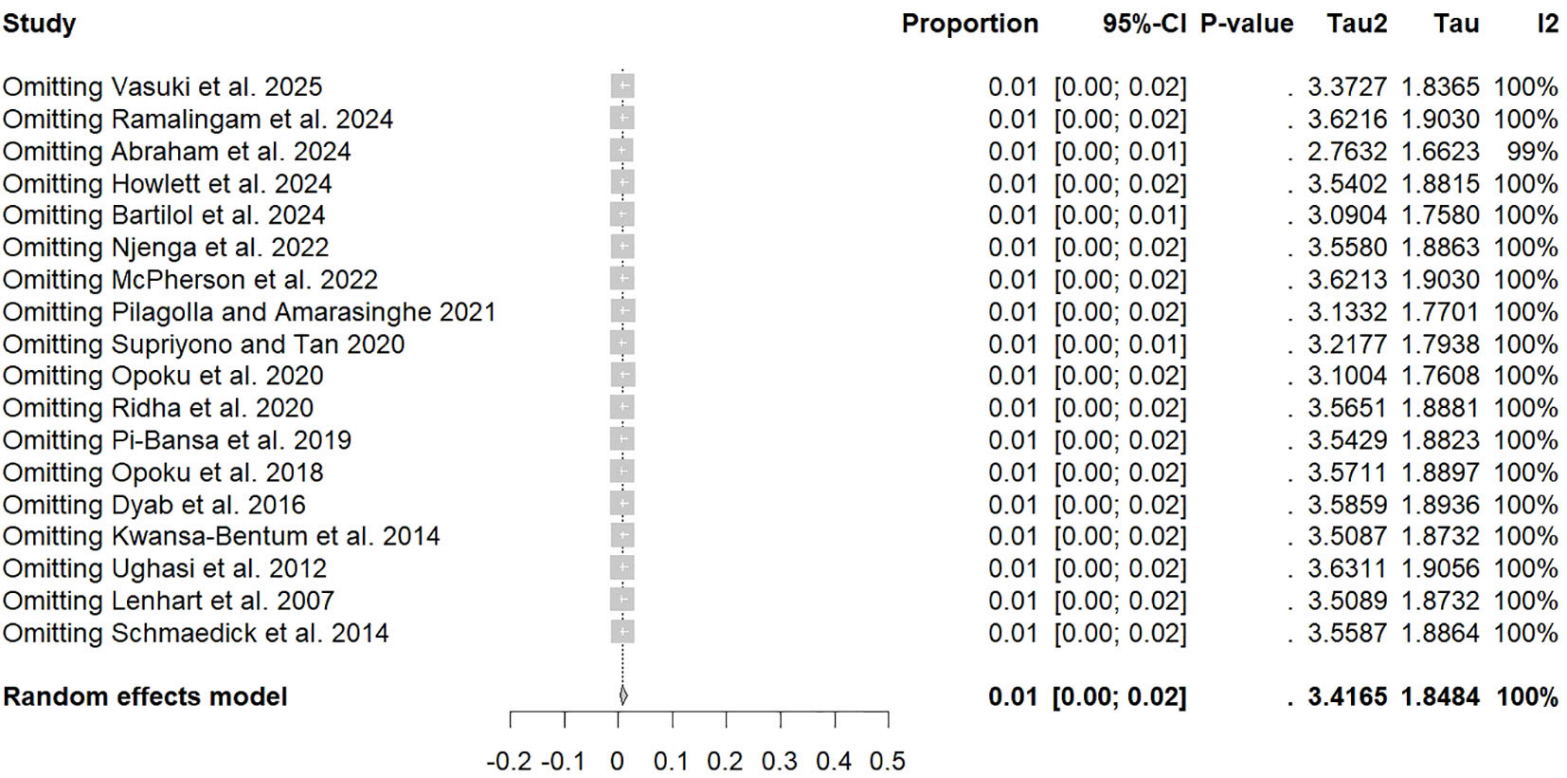
Sensitivity analysis.

Sensitivity analysis demonstrated that removal of individual studies did not substantially alter pooled prevalence estimates, indicating robust results (**Figure 5**). Among the four transformation methods tested, the logarithmic transformation (PLN) was selected because it provided the best approximation to a normal distribution based on the Shapiro-Wilk test (W value close to 1, *p* > 0.05), whereas the double arcsine method (PFT), although widely used in epidemiological studies, showed less satisfactory performance in this dataset (**Table 3**).

**Table 3:** Normal distribution test and different conversion of normal prevalence.

Conversion form	*Shapiro–Wilk*	*P–value*
PRAW	0.55798	0.0000
PLN	0.95975	0.5969
PLOGIT	0.95838	0.5707
PAS	0.74935	0.0003
PFT	0.75202	0.0003

“PRAW”: The Original rate; “PLN”: The Logarithmic conversion;

“PLOGIT”: The logit transformation; “PAS”: Arcsine transformation;

“PFT”: Double–arcsine transformation.

### 3.4 Meta-analysis results

The overall prevalence of filarial nematode infection in mosquitoes was estimated at 0.7% (1481/160,423; 95% CI: 0.3-1.7) across the 18 studies.

Regional analysis revealed that Asia exhibited the highest prevalence (3.0%, 95% CI: 0.0-10.7), while Oceania showed the lowest (0.3%, 95% CI: 0.2-0.4).

Analysis of seasonal subgroups revealed that the infection rates in samples gathered in winter (1.8%, 95% CI: 0.0-8.0) were greater than those collected in summer (1.2%, 95% CI: 0.3-2.7).

Temporal analysis showed that studies conducted after 2016 reported a higher prevalence (1.9%, 95% CI: 0.1-5.7) compared to those from 2016 or earlier.

The results were also affected by the sampling techniques used: the infection rate for mosquitoes gathered using CDC light traps was the highest at 4.3% (95% CI: 0.0-22.6).

Vector genus analysis revealed that *Mansonia* spp. had the highest prevalence (2.5%, 95% CI: 0.8-4.9), whereas *Culex* spp. had the lowest (0.1%, 95% CI: 0.0-0.3).

Diagnostic methods demonstrated varying sensitivity: RT-PCR detected the highest prevalence (2.5%, 95% CI: 0.0-11.0), while DM yielded the lowest (1.3%, 95% CI: 0.0-4.4).

Mass drug administration (MDA) implementation was associated with reduced prevalence; mosquitoes from areas without MDA programs showed higher infection rates (1.7%, 95% CI: 0.4-3.8).

Climatic subgroup analyses suggested that prevalence peaked in regions with mean annual temperatures of 23-27 °C (5.2%, 95% CI: 0.4-14.6) and mean annual precipitation of 500–1000 mm (10.7%, 95% CI: 0.0-41.8). By contrast, prevalence was lowest in areas with mean annual temperatures of 28-30 °C (0.4%, 95% CI: 0.1-0.8).

## 4 Discussion

This meta-analysis synthesizes current evidence on the prevalence of *lymphatic filariasis* (LF) parasites in mosquito vectors and highlights ecological, climatic, and methodological factors that influence transmission dynamics. Across 18 eligible studies involving 160,423 mosquitoes, an overall infection prevalence of 0.7% was observed, reflecting both the persistence of LF transmission and the challenges faced by elimination programs.

### 4.1 Geographic and vector-related differences

Analyses of subgroups revealed significant differences in the prevalence of infections across various geographic regions, showing that Asia had the highest rate at 3.0% (95% CI 0.0-10.7), while Oceania exhibited the lowest rate of 0.3% (95% CI 0.2-0.4). These differences can be explained by variations in vector species composition, ecological adaptations, and local climatic factors. Previous studies have shown that *Mansonia* spp. are the primary LF vectors in Southeast and South Asia (Pimnon et al., 2024; Saeung et al., 2015), whereas *Aedes* and *Culex* predominate in Oceania (Bhuvaneswari et al., 2023; Bohart and Ingram, 1946; Cameron and Ramesh, 2021; Russell and Burkot, 2023).Subgroup analysis of mosquito genera confirmed this pattern: *Mansonia* spp. exhibited the highest infection prevalence (2.5%, 95% CI 0.8-4.9), while *Culex* spp. showed the lowest (0.1%, 95% CI 0.0-0.3). The strong association between *Mansonia* spp. and aquatic vegetation, such as duckweed and water hyacinth, provides abundant breeding sites in Southeast Asia and Indonesia, contributing to high mosquito densities and increased infection rates (Pimnon et al., 2024). Moreover, the widespread use of artificial water storage in agricultural practices, including rice cultivation, further supports *Mansonia* breeding (Pratiwi et al., 2021; Richards et al., 2010). In contrast, the geographic isolation of Polynesian islands reduces vector dispersal and population density, thereby limiting LF transmission (Levin, 2014). Collectively, these findings underscore the influence of ecological context on vector competence and transmission intensity.

### 4.2 Climatic influences

The prevalence of infections is significantly influenced by climatic factors. An analysis of subgroups based on the mean annual temperature indicated that areas with average temperatures ranging from 23 to 27 °C exhibited the highest prevalence at 5.2% (95% CI 0.4-14.6), while the lowest prevalence was observed in regions with temperatures between 28 and 30 °C, recorded at 0.4% (95% CI 0.1-0.8). Prior studies indicate that filarial worm development accelerates with increasing temperature (Hu et al., 1995), but approximately 27 °C represents an optimal balance between development rate and mosquito survival (Fang et al., 2000; Li and Wang, 1992). At 25–28 °C, the mosquito intrinsic growth rate (*r_m_*, the maximum per capita rate of population increase under optimal environmental conditions) reaches its peak, maximizing transmission potential (Liu et al., 1994). In contrast, at temperatures above 31 °C, reduced survival, impaired oviposition, and smaller body size compromise mosquito fitness and transmission efficiency (Rueda et al., 1990).

Rainfall patterns demonstrated a similar influence. Areas with 500–1000 mm annual precipitation exhibited the highest prevalence (10.7%, 95% CI 0.0-41.8). Such environments provide stable aquatic habitats and favorable humidity, enhancing oviposition and larval development (Afrane et al., 2012; Ishak and Kasman, 2018). However, the wide confidence interval indicates considerable imprecision, likely resulting from limited data availability and methodological heterogeneity. Hence, these findings should be interpreted cautiously until validated by larger datasets. By contrast, precipitation exceeding 1000 mm may cause flooding, habitat disruption, and altered water quality, ultimately lowering vector abundance and infection prevalence (Dieng et al., 2012; Paaijmans et al., 2007; Tulak et al., 2018).

### 4.3 National and programmatic variation

At the country level, India exhibited the highest infection prevalence (4.8%, 95% CI 0.0-24.6), while Côte d’Ivoire (0.03%, 95% CI 0.0-0.1) and Samoa (0.3%, 95% CI 0.2-0.5) recorded the lowest. The limited data available for Côte d’Ivoire may have introduced selection bias, as only one study was included with very few positive samples. In contrast, multiple studies from Samoa consistently showed low prevalence. Despite India’s nationwide MDA program, coverage varied widely (48.8-98.8%), with significant disparities between urban and rural regions (Murthy and Kumar, 2018). The high mobility of the urban population and the complex structure of urban communities make it difficult to achieve high coverage through centralized distribution (Dinesh et al., 2023). The population’s adherence to MDA is low, especially in rural areas, owing to low economic and educational levels (Jakhar et al., 2022; Sekher, 2011). These factors explain the persistence of mosquito infections despite large-scale interventions. Conversely, Samoa successfully implemented triple-drug therapy with coverage exceeding 80%, supported by strong community mobilization and educational campaigns. The country’s small population facilitated high compliance rates, reaching 99% adherence, which effectively reduced mosquito infection prevalence (Willis et al., 2020). These contrasting outcomes illustrate that MDA success is strongly dependent on coverage, adherence, and programmatic context.

### 4.4 Seasonal and methodological factors

An analysis of seasonal patterns revealed that the prevalence was greater in winter (1.8%, 95% CI 0.0-8.0) than in summer (1.2%, 95% CI 0.3-2.7). This pattern may reflect seasonal extremes in endemic regions, where high summer temperatures (>30 °C) and heavy rainfall reduce mosquito survival and thereby interrupt transmission (Dieng et al., 2012; Paaijmans et al., 2007; Rueda et al., 1990).

Methodological differences further influenced reported prevalence. RT-PCR yielded the highest infection estimates (2.5%, 95% CI 0.0-11.0), while dissection microscopy yielded the lowest (1.3%, 95% CI 0.0-4.4). Although RT-PCR showed higher sensitivity, the difference across diagnostic methods was not statistically significant in the meta-regression model (p > 0.05), likely due to limited study numbers and heterogeneity. Although microscopy has historically been the gold standard, molecular assays such as PCR and RT-PCR detect submicroscopic infections and are increasingly preferred for surveillance in post-MDA settings (Derua et al., 2017). Similarly, CDC light traps were associated with higher infection prevalence (4.3%, 95% CI 0.0–22.6) than alternative sampling techniques. This trapping method offers advantages that may explain its superior performance. Unlike human landing catches or resting collections, which rely on operator skill, limited sampling windows, and potential ethical concerns, CDC miniature light traps operate continuously overnight and standardize mosquito attraction using controlled cues such as carbon dioxide release and ultraviolet illumination (Namango et al., 2022). These stimuli effectively mimic host-seeking signals and enhance the capture of nocturnal mosquito species that are primary lymphatic filariasis vectors (Abong'O et al., 2021). In contrast, ovitraps and indoor aspiration tend to target specific behavioral subsets of mosquitoes and may underrepresent actively blood-seeking females (Tsunoda et al., 2020). Therefore, the ability of CDC miniature light traps to capture a broader and more epidemiologically relevant subset of vector populations likely contributes to their higher observed infection rates (Ahmad et al., 2016; Amuzu et al., 2010).

### 4.5 Temporal trends

Studies conducted after 2016 reported higher estimated prevalence rates compared to earlier studies. This increase is likely attributable to improved diagnostic sensitivity and broader use of molecular assays following the launch of the GPELF (Global Programme to Eliminate *lymphatic filariasis*) strategy (Addiss and Global, A.T.E.L, 2010). The wider availability of PCR equipment, reduced reagent costs, and standardization of protocols have enhanced surveillance capacity, particularly in resource-limited settings (Anjum et al., 2018; Ince and McNally, 2009; Kuypers et al., 2009; Zhao et al., 2020). Molecular detection methods, especially RT-PCR, have revealed infections that were previously undetectable by traditional dissection, including submicroscopic infections (Bartilol et al., 2024a; Bartilol et al., 2024b; Haanshuus et al., 2016). Thus, the apparent post-2016 increase is most likely attributable to methodological advances, although localized resurgence in certain regions cannot be entirely excluded.

### 4.6 Limitations

This study has certain limitations. To begin with, the pool of eligible studies was relatively small, and signs of publication bias were detected. Second, insufficient data were available for more refined subgroup analyses, including species-specific infection rates and sex-specific infection rates in mosquitoes. Third, many studies scored low on quality assessments (Munn et al., 2015; Porritt et al., 2014), largely due to inadequate reporting of sampling details. These issues highlight the need for more comprehensive study designs and standardized reporting practices in future research.

## 5 Conclusion

In conclusion, this systematic review and meta-analysis provides the first comprehensive synthesis of mosquito infection rates with filarial nematodes across multiple regions and surveillance methodologies. The findings demonstrate clear geographic and ecological variation, with *Mansonia* spp. contributing disproportionately to transmission in Asia, while prevalence remains low in Oceania. Climatic conditions, sampling approaches, and diagnostic techniques also significantly influenced reported prevalence, underscoring the complexity of LF transmission dynamics. Crucially, the findings emphasize that the successful execution of mass drug administration (MDA), bolstered by robust community involvement and health system capabilities, is fundamental to decreasing mosquito infection rates and halting transmission.

Future research should prioritize standardized surveillance methods, integrate molecular diagnostics with traditional entomological tools, and expand data collection in underrepresented regions. These initiatives will not only enhance the oversight of elimination programs but will also offer essential perspectives for maintaining long-term management and reaching the worldwide objective of eradicating LF as a public health issue.

## Data Availability

The original contributions presented in the study are included in the article/**Supplementary Material**. Further inquiries can be directed to the corresponding authors.
